# Core Altered Microorganisms in Colitis Mouse Model: A Comprehensive Time-Point and Fecal Microbiota Transplantation Analysis

**DOI:** 10.3390/antibiotics10060643

**Published:** 2021-05-28

**Authors:** Lijun Shang, Hongbin Liu, Haitao Yu, Meixia Chen, Tianren Yang, Xiangfang Zeng, Shiyan Qiao

**Affiliations:** 1 State Key Laboratory of Animal Nutrition, Ministry of Agriculture Feed Industry Centre, China Agricultural University, Beijing 100193, China; shanglj1996@163.com (L.S.); binhongliu@126.com (H.L.); 15600660793@163.com (H.Y.); meixia10nian@163.com (M.C.); yangtr@cahg.com.cn (T.Y.); ziyangzxf@163.com (X.Z.); 2 Beijing Bio-Feed Additives Key Laboratory, Beijing 100193, China

**Keywords:** colitis, microbiota, microbiota transplantation, *Muribaculaceae*, *Turicibacter*, *Lachnospiraceae*

## Abstract

Inflammatory bowel disease (IBD), including Crohn’s disease (CD) and ulcerative colitis (UC), is characterized by chronic and relapsing inflammation within the gastrointestinal tract. Antibiotics have been used to treat IBD, primarily utilizing metronidazole. Although there does seem to be a treatment effect, the broad-spectrum antibiotics that have been used to date are crude tools and have many adverse effects. Available evidence suggests that the host microbiome is implicated in the pathogenesis of IBD, though the key bacteria remain unknown. If the bacterial population can be modified appropriately, the use of antibiotics will have a better therapeutic effect. In this study, mice were fed dextran sodium sulfate (DSS) solution for 5 days, followed by 5 days of normal drinking water, to investigate the gut microbiota response to colitis and the initial alteration of microbiota in recovery phase. Day 0 was considered the normal control, while day 5 and day 10 were considered the colitis mouse model progressive phase and recovery phase, respectively. Results showed that inflammation could induce proportional changes in the gut microbiota. Furthermore, transplanting the microbiota in progressive phase to antibiotic-induced microbiota-depleted mice could induce inflammation similar to colitis, which proves the importance of initial alteration of the microbiota for IBD recovery and the potential of the microbiota as a target for the treatment of IBD. Meanwhile, we have also identified three possible target microorganisms in the development of colitis, namely genera *Muribaculaceae* (negative correlation), *Turicibacter* (positive correlation) and *Lachnospiraceae* (negative correlation) in inflammation status through comprehensive analysis.

## 1. Introduction

The prevalence of inflammatory bowel disease (IBD), including ulcerative colitis (UC) and Crohn’s disease (CD), remains high in Europe and continues to rise across the world [1,2]. The former is confined to the colon, while the latter may affect any part of the digestive tract, with unclear mechanisms for the etiology. Based on the evidence from studies with IBD mouse models, especially the dextran sodium sulfate (DSS)-induced IBD mouse model, we know that bacteria are necessary for the development of IBD [3,4,5,6]. Regardless of the inconsistent observations regarding the microbial compositions of such patients, the observed dysbiosis is likely to contribute to disease severity [7,8].

Antibiotics have been used to treat IBD; primarily metronidazole [9,10,11], followed by ciprofloxacin [12] and amoxicillin [13], or cocktails of antibiotics [13,14]. The application of antibiotics decreases luminal bacterial concentration and may alter the composition of the gut microflora, favoring “beneficial” bacteria [15,16,17]. Antibiotics have also been shown to cause changes in microbial metabolism, with an increase in short-chain fatty acids and aromatic organic compounds and a decrease in glutamate and other compounds [16], which are directly related to the clinical response in IBD patients [16,18]. Unfortunately, although there does seem to be a treatment effect, the broad-spectrum antibiotics that have been used to date are crude tools and only associated with a moderate improvement in clinical symptoms [19]. Existing data on the role of antibiotics as a treatment strategy for IBD are inconsistent, leading to a limited role for antibiotics in clinical practice [20]. On the other hand, limited understanding of the microorganisms critical to the development of IBD has led to the direct and crude use of broad-spectrum antibiotics. A better understanding of host–microbial interactions in physiological as well as disease settings may lead to the development of antibiotic treatments.

Numerous studies have identified the compositional changes of microbiota that result from DSS administration. However, inconsistent observations regarding the microbial composition of such disease models have hindered assessment of the role of specific bacterial species in the pathophysiology of IBD. This may be due to the model being at different stages of the disease. In the present study, we used oral DSS solution for 5 days to establish a colitis mouse model and selected mice from random cages during sampling to reduce any cage effect. Colonic content samples were collected on day 0 (d 0) and day 5 (d 5), and the response of the gut microbiota to colitis was investigated in combination with the latest research. After a 5-day recovery period, colonic content samples were collected on day 10 (d 10) to investigate initial changes in microbiota during the recovery period. To clarify the uncertainty about the specific function of inflammation-induced microbiota alterations, we transplanted colonic bacteria from the d 5 model to Abx mice. FMT mice showed symptoms similar to those of the colitis mouse model, indicating a shift in the intestinal flora from a normal symbiont to a harmful pathogenic state, and may elucidate the cause of relapse in IBD patients. Additionally, based on the correlation between the bacterial community and three important inflammatory parameters, combined with the key microbiota leading to colitis-related symptoms in mice after FMT, *Muribaculaceae*, *Turicibacter* and *Lachnospiraceae* have been identified as key targets for microbiota changes in colitis. This may be the key to understanding the different roles of microbiota at different stages of IBD and reveal new therapeutic intervention strategies. Our aim is to highlight the importance of selecting appropriate antibiotics when investigating the treatment of colitis based on gut microbiota regulation, and the value of the knowledge generated by such in vivo tests to translation in humans in the future.

## 2. Materials and Methods

### 2.1. Animals

Six- to eight-week-old female C57BL/6J mice used in the present study were purchased from SPF (Beijing, China) Biotechnology Co., Ltd. Mice were housed under the same conditions (specific pathogen-free conditions: temperature, 24 ± 1 °C; lighting cycle, 12 h:12 h light/dark) and had free access to food and drinking water. Animal experiments were performed in accordance with the Animal Care and Use Committee of China Agricultural University (Beijing, China).

### 2.2. Mouse Models

**Figure 1:**
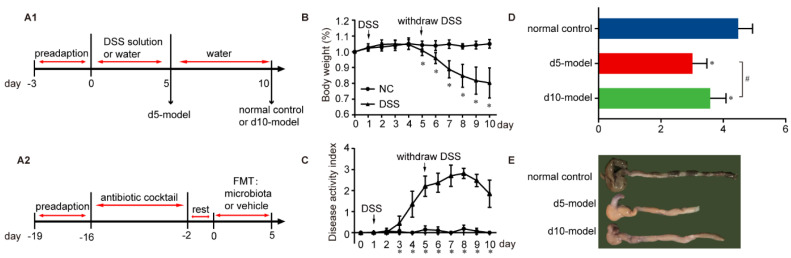
Clinical signs of IBD mouse model. (**A1**) Experimental design of time-dependent microbial changes in colitis. (**A2**) Experimental design of microbiota transplantation. (**B**) Body weight changes (relative to original weight, set as 100%) and (**C**) DAI over the whole experimental period. NC, *n* = 9; DSS, D 0 to d 5, *n* = 18; d 6 to d 10, *n* = 9. Differences were determined via a two-way ANOVA followed by a Sidak’s multiple comparisons test. (**D**) Length of the colon between the ileocecal junction and the proximal rectum, *n* = 9. Data were analyzed by one-way ANOVA, followed by Tukey’s multiple comparisons test, *n* = 9. (**E**) The representative pictures of colon. All of the data are expressed as the mean ± SD. * *p* < 0.05 compared with the normal control group; # *p* < 0.05 compared with the d 5 model group and the same below.

A total of 27 eight-week-old female C57BL/6J mice were provided ad libitum access to drinking water with 5% DSS (36–50 kDa; MP Biomedicals, Irvine, CA, USA) for 5 days to establish a colitis mouse model. They then received normal drinking water for an additional 5 days. A total of 9 eight-week-old female C57BL/6J mice had no treatment throughout the whole experiment to set a normal control. The mice were euthanized by CO_2_ asphyxiation and blood, colon tissue and colonic contents of the colitis mouse model were collected on day 5 (d 5 model, *n* = 9; donor mice for microbiota transplantation experiment, *n* = 9) and day 10 (d 10 model, *n* = 9); colon tissue and colonic contents of the normal control were collected on day 10. (Figure 1A). Body weight, stool consistency and degree of intestinal bleeding were measured daily. The scoring system displayed in Table S1 was described by Wirtz et al. [6].

For the antibiotic-induced microbiota depletion mouse model (Abx mice), we used the methods as described previously with slight modification [21,22,23,24,25]. Six-week-old female C57BL/6J mice were provided a cocktail containing ampicillin, neomycin, metronidazole and vancomycin (1 g/L, 1 g/L, 1 g/L and 500 mg/L, respectively and all were obtained from Sigma, St. Louis, MI, USA) in drinking water ad libitum for 2 weeks, and given “rest” for 2 days to prepare for microbiota transplantation (Figure 1A).

### 2.3. Microbiota Transplantation

Fresh colonic content samples were collected from donor mice (colitis mouse model on day 5, *n* = 9) and homogenized in sterile phosphate buffer saline (PBS, Beijing Solarbio Science & Technology Co., Ltd., Beijing, China, 50 mg/mL). Homogenates were passed through a 40 µm cell strainer and then centrifuged at 8500 rpm for 5 min. Precipitation was resuspended with the same volume of 10% glycerol/PBS solution and stored at −80 °C until used for microbiota transplantations.

Five Abx mice were intragastrically administered with a 200 μL suspension once a day for 5 consecutive days (FMT mice), while 5 mice were intragastrically administered with the same volume of 10% glycerol (Beijing Solarbio Science & Technology Co., Ltd., Beijing, China)/PBS vehicle as the control (vehicle mice). Mice were then euthanized, and colon tissue and colonic contents were collected. Body weight, stool consistency and degree of intestinal bleeding were measured daily.

### 2.4. ELISA

Blood (collected on day 0, day 5 and day 10 of the IBD mouse model) was centrifuged to obtain serum. The levels of IFN-γ, IL-1β and TNF-α were determined using ELISA kits (SLCY Biotech, Beijing, China). The kit assays were carried out according to the protocol supplied by the manufacturer. The absorbance was read at 450 nm using a multimode microplate reader (iMark, BIORAD, Hercules, CA, USA).

### 2.5. Histology

Colon sections were removed, the contents were gently extruded and then washed in saline, fixed in 4% paraformaldehyde and embedded in paraffin. After being cut into 4 μm thick slices, tissue sections were stained with hematoxylin and eosin and then examined using a light microscope (Nikon Eclipse Ci, Tokyo, Japan). Photomicrographs were captured using a digital camera attached to the microscope (Nikon Digital Sight DS-FI2, Tokyo, Japan). Histological damage was quantitatively assessed as described by Wirtz et al. [6]. The sum of two sub-scores resulted in a combined score ranging from 0 (no changes) to 6 (widespread cellular infiltrates and extensive tissue damage) (Table S2).

### 2.6. Microbiota Composition by 16S rRNA Sequencing Analysis

Colonic contents were collected from the colitis mouse model on day 0, day 5 and day 10. Genomic DNA was extracted using the E.Z.N.A.^®^ Soil DNA Kit (Omega Bio-tek, Norcross, GA, USA). The V3-V4 hypervariable regions of the bacterial 16S rRNA gene were amplified with primers 338F (5′-ACTCCTACGGGAGGCAGCAG-3′) and 806R (5′-GGACTACHVGGGTWTCTAAT-3′) by a thermocycler PCR system (GeneAmp 9700, ABI, Vernon, CA, USA). Purified amplicons were pooled in equimolar concentrations and paired-end sequenced (2 × 300) on an Illumina MiSeq platform (Illumina, San Diego, CA, USA). Extract all sample sequences according to the smallest sequence number sample so that the sequence number of all samples are the same. Raw FASTQ files were demultiplexed and quality-filtered using QIIME (version 1.17). Operational taxonomic units (OTUs) were clustered with 97% similarity by UPARSE62 (version 7.1 http://drive5.com/uparse/, 26 December 2019) and chimeric sequences were identified and removed using UCHIME. The taxonomy of each sequence was analyzed by RDP Classifier (http://rdp.cme.msu.edu/, 26 December 2019) against the Silva (SSU115) 16S rRNA database with a 70% confidence threshold [26].

### 2.7. Statistical Analysis

Statistical analysis was performed using Prism9 software (GraphPad Software Inc., San Diego, CA, USA). Data were first checked for normal distribution and plotted in the figures as mean ± SD. For each figure, *n* = the number of independent biological replicates. No samples or animals were excluded from the analyses. For experiments containing more than two relative groups, one-way ANOVA followed by Dunnett’s or Tukey’s multiple comparisons test was performed. Differences in bacterial data were evaluated by the Wilcoxon rank sum test or Kruskal–Wallis H test (the choice of specific method will be explained again under the figures), and correlation study used Spearman analysis. Data were expressed as mean ± SEM, *p* values < 0.05 were considered statistically significant.

## 3. Results

### 3.1. Clinical Signs

**Figure 2:**
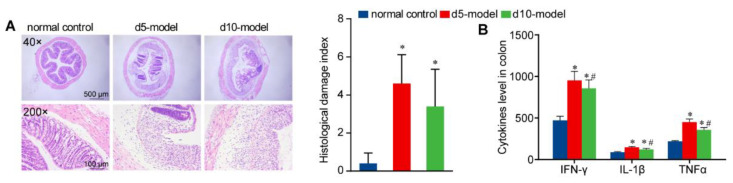
Inflammatory conditions of IBD mouse model. (**A**) Representative images of the colon by H&E staining (40× and 200×). (**B**) Score of inflammation-associated histological changes in colon. (**B**) The concentration of IFN-γ, IL-1β and TNF-α in serum, *n* = 9. Data are means ± SD and analyzed by a one-way ANOVA followed by Tukey’s multiple comparisons test. * *p* < 0.05 compared with the normal control group; # *p* < 0.05 compared with the d 5 model group.

After 5 days of DSS treatment, the colitis mouse model showed a significant decrease in body weight, with continued decline for the next 5 days, but did not show a significant downward trend compared with the previous day from day 8 (Figure 1B). Disease activity index began to rise significantly on the 3rd day and peaked on day 8 (Figure 1C). Colon length was decreased compared with the normal control (Figure 1D,E). We also noted clear signs of edema, severely damaged mucosal structures and abundant inflammatory cell infiltration via H&E staining of the distal colon tissue (Figure 2A). Serum concentrations of the inflammatory cytokines IFN-γ, IL-1β and TNF-α significantly increased (Figure 2B). It is worth noting that, compared with d 5 model, the colon length of the d 10 model significantly increased whereas the serum inflammatory cytokines significantly decreased.

### 3.2. Gut Microbiota Altered in Colitis Mouse Model

**Figure 3:**
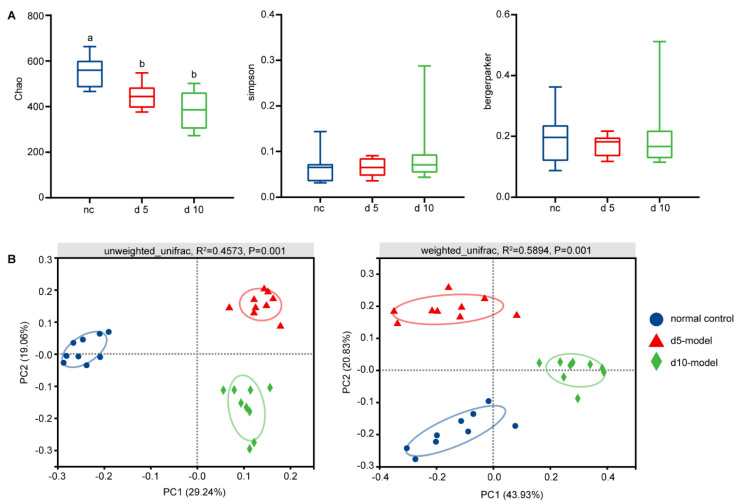
The changes in gut microbiota diversity. (**A**) Alpha diversity changes. (**B**) Principal coordinate analysis by unweighted UniFrac (**left**) and weighted UniFrac method (**right**), *n* = 9. Data are compared using Kruskal–Wallis tests with Benjamini–Hochberg correction.

As rodents are coprophagic, the gut microbiota of mice in the same cage will progressively become homogeneous over time. In order to avoid this, mice were randomly selected from multiple cages each time for sample collection. Significantly decreased richness diversity, assessed by Chao, were noted across the d 5 and d 10 models (Figure 3A). However, no differences in Simpson and Berger–Parker dominance indices were noted between groups, which indicated that the abundance of dominant species in each group was basically the same. (Figure 3A).

Additionally, PCoA revealed clear clustering based on both unweighted UniFrac and weighted UniFrac metrics (Figure 3B; *p* = 0.001, PERMANOVA), indicating that detected community differences were not due to the presence and/or absence of rare taxa.

### 3.3. Correlation Analysis between Specific Microbiota and Disease Indicator

**Figure 4:**
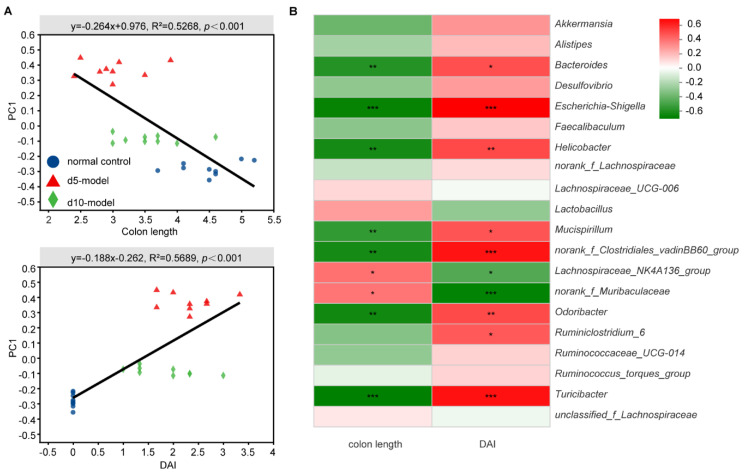
Correlation between specific microbiota and disease indicators. (**A**) Linear regression relationship between microbiota and inflammation-associated parameters. (**B**) Correlation matrix between specific microbiota genera and inflammation-associated parameters (Spearman correlation). * *p* < 0.05, ** *p* < 0.01, *** *p* < 0.001, means the correlation between horizontal and vertical coordinates is significant.

To further identify the key microorganisms, correlation analysis was used. Among three important inflammation-associated parameters, disease activity index and colon length were highly correlated with microbial structure by linear regression analysis (Figure 4A and Supplementary Figure S1).

More concretely, Bacteroides, Escherichia-shigella, Helicobacter, Mucispirillum, norank_f_Clostridiales_vadinBB60_group, Odoribacter, Ruminiclostridium and Turicibacter were significantly positively correlated with inflammation-associated parameters, while Lachnospiraceae_NK4A136_group and norank_f__Muribaculaceae were significantly negatively correlated with inflammation-associated parameters (Figure 4B).

### 3.4. Colitis Mouse Model Colonic Commensal Microbiota Contributed to Colitis

**Figure 5:**
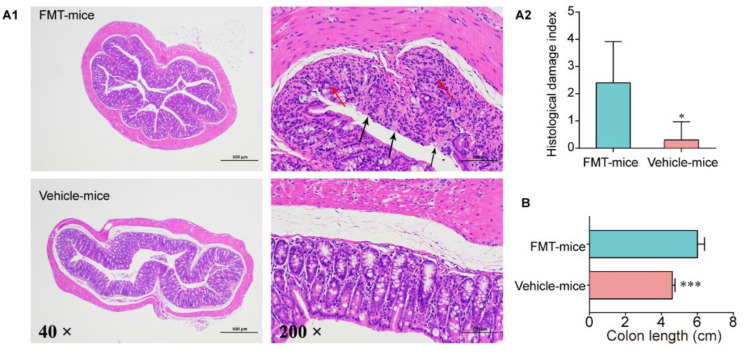
Inflammatory conditions of the colon after FMT. (**A1**) Representative images of the colon by H&E staining (40× and 200×). The red arrow indicates morphological changes of mucous layer, and the black arrow indicates edema status in submucosa. (**A2**) Score of inflammation-associated histological changes in the colon. (**B**) Colon length after FMT, *n* = 5. Data are means ± SD and analyzed by Student’s t-test. * *p* < 0.05, *** *p* < 0.0001.

In order to further define the pathogenicity of the intestinal flora in the colitis progressive phase and to narrow the range of species that play an important role in colitis, FMT was performed. Compared with vehicle mice, colon length of FMT mice decreased significantly (Figure 5B and Figure S2), and some colon samples of FMT mice showed obvious blood and hygromata after microbiota transplantation (Figure S2). However, body weight was not significantly different and no obvious sign of blood was observed in feces (data not shown). Similarly, mucosal epithelial damage and epithelial loss were observed in almost all FMT mice, and we detected obvious inflammatory infiltration in a few FMT mice (Figure 5A). 

### 3.5. Microbial Diversity Changes after FMT

**Figure 6:**
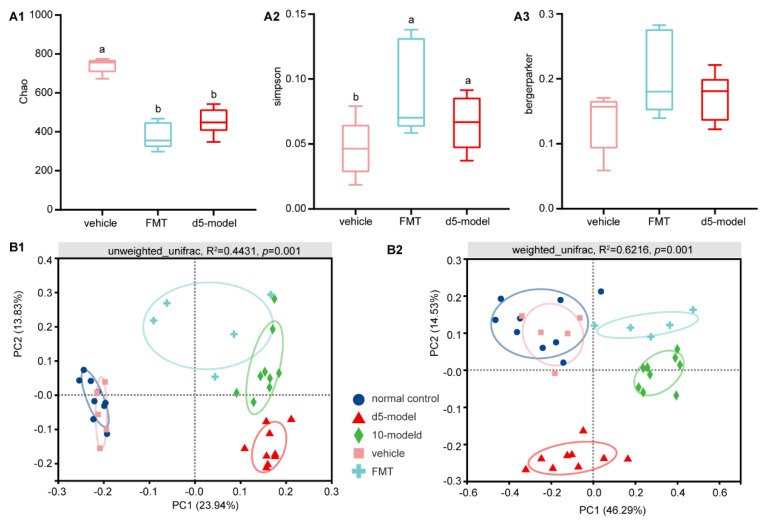
The changes in gut microbiota diversity after FMT. (**A**) Alpha diversity changes. (**B**) Principal coordinate analysis by unweighted UniFrac (**left**) and weighted UniFrac method (**right**), *n* = 5. Data are compared using Kruskal–Wallis tests with Benjamini–Hochberg correction, different lowercase letters within each group indicate significantly different values.

In addition to colitis symptoms in FMT mice, community evenness and richness diversity significantly decreased compared with the vehicle mice (Figure 6A). However, no differences in the Berger–Parker dominance index were noted (Figure 6A). Similarly, PCoA revealed clear clustering based on both unweighted UniFrac and weighted UniFrac metrics (Figure 6B; *p* = 0.001, PERMANOVA).

### 3.6. Key Microorganisms Associated with Colitis

**Figure 7:**
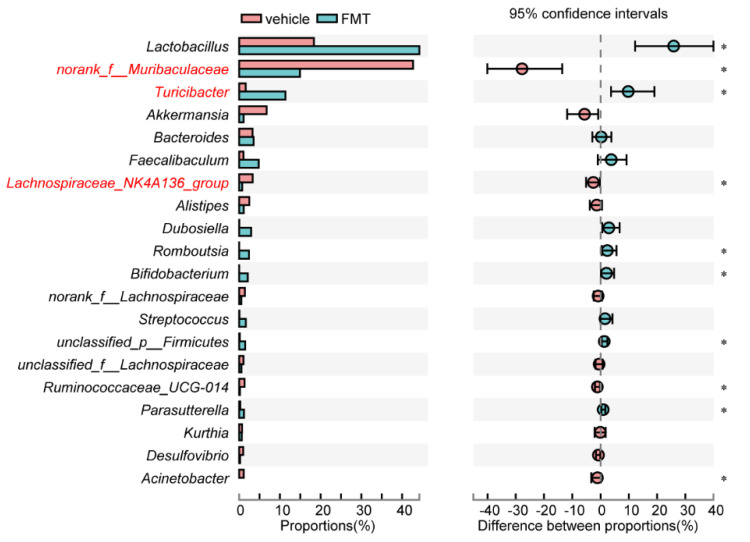
Relative abundances of the top 20 genera. The red font indicates there was also a significant difference in the Figure 4 association analysis of the previous experiment. * *p* < 0.05.

The most differential microorganisms between FMT and vehicle mice are shown in Figure 7, three of which were also significantly correlated with inflammation-associated parameters. More concretely, the decreased *norank_f_Muribaculaceae* and *Lachnospiraceae_NK4A136_group*, as well as the increased *Turicibacter*, contributed to colitis development.

## 4. Discussion

The indigenous gut microbiota is believed to play a key role in the pathogenesis of inflammatory bowel disease. Much of the evidence for the involvement of gut microbiota in IBD comes from studies with both murine models of disease and with human patients [6,27,28,29]. Although numerous studies have identified the compositional changes of microbiota, inconsistent observations regarding the microbial compositions of such disease models have hindered assessment of the role of specific bacterial species in the pathophysiology of IBD. These inconsistent observations may be caused by the model at different stages of the disease. On the other hand, although many studies have reported the alterations in gut microbiota composition during acute colitis, the recovery from dysbiosis has received little attention. Hence, we screened the responses of colon bacterial flora to DSS-induced colitis and the initial alterations of microbiota from the progressive phase to the recovery phase. Transplanting microbiota technology was used to revalidate the role of altered microbiota in pathogenicity.

After 5 days of DSS treatment, the significant weight loss, increased DAI and reduced colon length that was observed in the present study was in agreement with data reported previously [6,30], which indicated that the IBD mouse model was successfully established. Additionally, DSS-treatment significantly reduced Chao index, indicating that the richness of microflora decreased. Similarly, many previous studies have reported that the diversity of the intestinal microbiota of IBD patients decreased compared to that of healthy controls [3,31,32]. However, we did not observe significant changes in evenness and abundance of dominant species. On the other hand, PCoA revealed clear clustering based on both unweighted UniFrac and weighted UniFrac metrics, indicating that dominant species are different in the healthy controls, the d 5 model and the d 10 model.

Many previous studies have shown that shortened colon length and increased DAI were important inflammation-associated parameters in DSS-induced colitis mouse models [3,6,27,30]. In the present study, significant correlation between the structure of the bacterial community with colon length and DAI provides evidence for the close connection between bacterial community structure and disease severity.

However, the specific function of microbiota alterations induced by inflammation remains unclear. To clarify this uncertainty, we transplanted colonic bacteria from the IBD d 5 model to Abx mice. As a result, FMT mice showed symptoms similar to those of the IBD mouse model while vehicle mice remained healthy. This result implies that gut bacterial flora in the IBD mouse shifts from normal commensal flora to harmful pathogenicity status and potentially elucidated the relapse of IBD. On this basis, one taxon positively correlated with disease severity and two taxa negatively correlated with disease severity were screened out. *Muribaculaceae* abundance was strongly correlated with propionate [33], which can inhibit the CD8^+^ T cell activation to tolerate the immunity stimulation [34]. This may explain the present negative correlation between *Muribaculaceae* with inflammation status. Similar to the present study, members of *Lachnospiraceae* have been linked to protection from colon inflammation in humans, mainly due to the association of many species within the group with the production of butyric acid [35], a substance that is important for both microbial and host epithelial cell growth [36,37,38]. Its production could also prevent the growth of some microbes within the digestive tract [39,40]. Past studies have suggested that *Turicibacter* may play a role in the development of IBD [41,42], which is also demonstrated in our study that *Turicibacter* has a strong positive association with inflammation.

Although this is very important, there is not enough clinical evidence to draw clear conclusions or recommendations for antibiotic preferences based on gut microbiota. The present experiment provides preliminary evidence of three possible targets in colitis development, which may be key to understanding the role of microbiota in colitis and revealing new strategies for therapeutic intervention, such as achieving a better therapeutic effect with the use of antibiotics.

This study does have limitations. Firstly, the exact mechanism between inflammation and the microorganisms that are key to the inflammatory state still needs to be investigated. Secondly, finding antibiotic targets that impact on the gut microbiome to alter the course of IBD makes good sense, but mouse models may be limited in their ability to represent human clinical trials. Consequently, further research is needed in the future and should be undertaken in the context of rigorously performed controlled trials to ensure that the interventions are truly effective and well tolerated.

## 5. Conclusions

This study revealed microbioal characteristics in different stages of colitis, and showed that *Muribaculaceae*, *Turicibacter* and *Lachnospiraceae* may be the key to the development of colitis among the complex changes. Meanwhile, transplanted colonic microbiota conferred colitis symptoms in Abx-mice, indicating that the refractory microbiota may be the cause of IBD recurrence. These factors must be taken into account in the future clinical use of antibiotics.

## Supplementary Materials

The following are available online at https://www.mdpi.com/article/10.3390/antibiotics10060643/s1, Figure S1: Linear regression relationship between microbiota and body weight, Figure S2: Pictures of colon after FMT, Table S1. Scoring system for calculating disease activity index (DAI), Table S2. Scoring system for inflammation-associated histological changes in the colon.

## Abbreviations

IBD	Inflammatory bowel disease
OTUs	Operational taxonomic units
CD	Crohn’s disease
UC	Ulcerative colitis
FMT	Fecal microbiota transplantation
SCFAs	Short-chain fatty acids
