# Supplementary material for: Core Altered Microorganisms in Colitis Mouse Model: A Comprehensive Time-Point and Fecal Microbiota Transplantation Analysis
Source: Antibiotics (Basel). 2021 May 28;10(6):643. doi: 10.3390/antibiotics10060643 (PMC8230101; doi:10.3390/antibiotics10060643)
Supplement: Supplementary file 1 [file antibiotics-10-00643-s001.zip › antibiotics-1204223-Supplementary.pdf]

antibiotics

MDPI

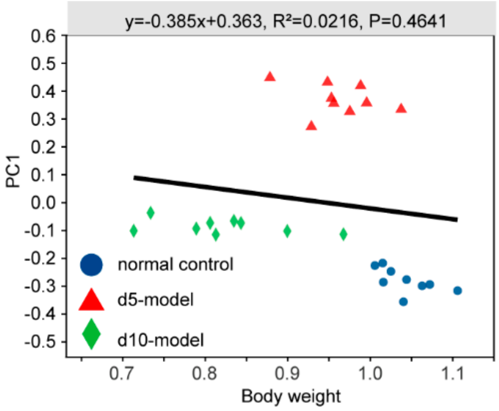

Figure S1. Lineal regression relationship between microbiota and body weight.

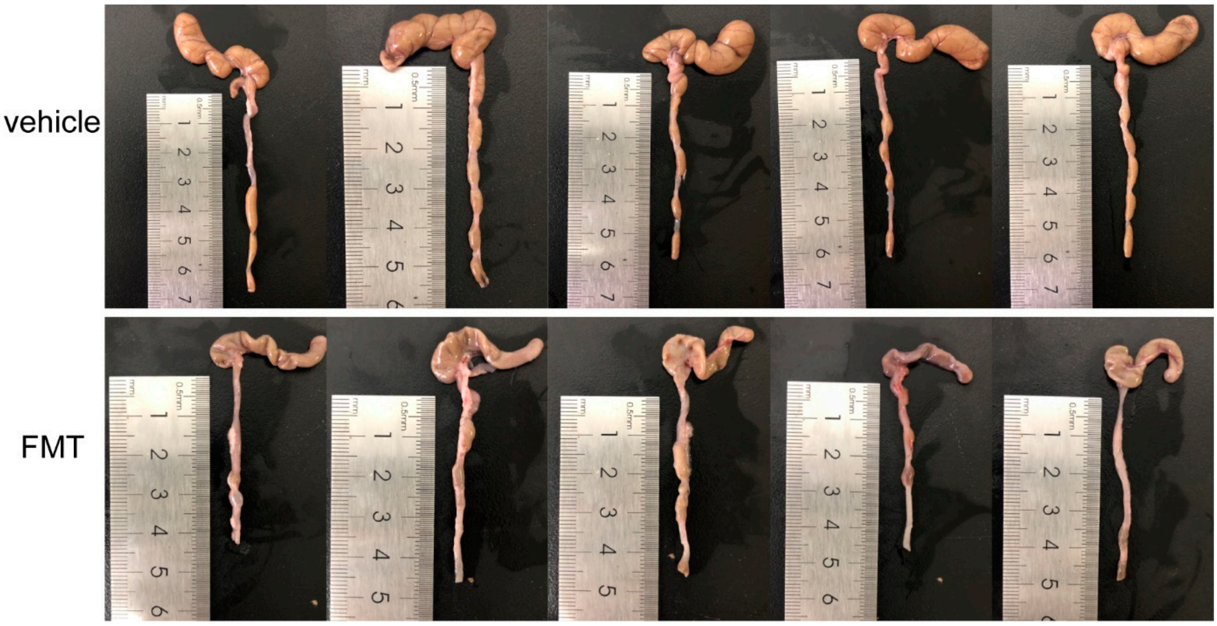

Figure S2. Pictures of colon after FMT.

Table S1. Scoring system for calculating disease activity index (DAI).

Score	Weight Loss	Stool Consistency	Blood
0	none	normal	negative hemoccult
1	1–5%	soft but still formed	negative hemoccult
2	6–10%	soft	positive hemoccult
3	11–18%	very soft; wet	blood traces in stool visible
4	>18%	watery diarrhea	gross rectal bleeding

Table S2. Scoring system for inflammation-associated histological changes in the colon.

Score	Tissue Damage	Lamina Propria Inflammatory Cell Infiltration
0	none	infrequent
1	isolated focal epithelial damage	increased, some neutrophils
2	mucosal erosions and ulcerations	submucosal presence of inflammatory cell clusters
3	extensive damage deep into the bowel wall	transmural cell infiltrations
